# Strategic priorities for reproducibility reform

**DOI:** 10.1371/journal.pbio.3001943

**Published:** 2023-01-12

**Authors:** Tony Ross-Hellauer

**Affiliations:** Know-Center GmbH and TU Graz, Graz, Austria

## Abstract

Increasing the reproducibility of research should be a top priority. This Perspective argues that more work is needed to combine efforts and maximize our actions to enable true reproducibility reform.

## Teaser

Increasing the reproducibility of research should be a top priority. Great work is being done, but more work is needed to combine efforts and maximize our actions to enable true reproducibility reform.

Recent years have stress-tested the scientific system. The COVID-19 pandemic demonstrated the potential for Open Science to aid humanity in rapid, collective action to meet catastrophic challenges [1]. But it also cruelly exposed the consequences of a continuing lack of societal trust in science (e.g., “anti-vax” sentiment) and, along with geopolitical unrest, has wrought economic havoc that will squeeze research funding in the coming years.

The specter of a “reproducibility crisis” has haunted meta-science and research policy conversations for years now [2]. Definitions vary, but at its broadest, reproducibility just means obtaining consistent results when repeating experiments and analyses. It is usually taken as a key tenet of science itself, if not a direct proxy for quality and credibility of results. Tackling the causes of poor levels of reproducibility stands to boost trust, integrity, and efficiency in research. Given the current circumstances, this should be a major priority for all research stakeholders, including funders, institutions, publishers, and individual researchers themselves.

**Fig 1:**
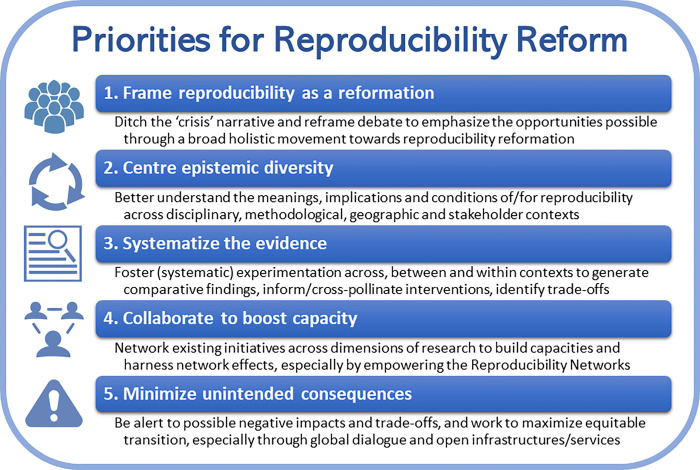
Priorities for reproducibility reform. Brief summary of five proposed strategic priorities to boost reproducibility research in ways which unite efforts, recognize epistemic differences, build an effective evidence base, harness network effects, and minimize unintended consequences. CC BY Tony Ross-Hellauer.

Much valuable work has already been done, but in my view, much of what we know, as well as the actions we are taking, are targeted narrowly on specific fields, with piecemeal initiatives and limited alignment of strategic action across stakeholders and elements of research. For broader reproducibility reform to take place and achieve maximum impact, I propose five strategic priorities for action (Fig 1).

## 1. Frame reproducibility as a reformation, not a crisis

The “crisis” narrative is unhelpful at best [3], if not just plain factually wrong [4]. While the crisis framing has been useful in alerting funders, institutions, and others to the importance and urgency of this issue, its overly dramatic tone should be replaced. Munafò and colleagues [3] argue that it is better to frame reproducibility as an “opportunity.” While I agree with the sentiment, I would argue that we should mobilize efforts through a shared image of what this opportunity means for us all. Hence, I prefer to conceptualize this work as a progressive movement for reform of institutions proven ill-suited for the challenges of our digital age, a “reproducibility reformation” if you will. However, treating reproducibility as a “whole system” issue in this way will require further work to better understand the meanings, causes, and implications of reproducibility across all aspects of the research system.

## 2. Center “epistemic diversity”

Concerns of a reproducibility crisis originated in disciplines such as psychology and clinical medicine, which constitute a narrow slice of the research spectrum. Much of what we know derives from those contexts [5]. Although other disciplines are increasingly alert to issues of reproducibility, collaborative action requires more work to understand differences and similarities in the causes and solutions to reproducibility across these contexts. Sabina Leonelli [6] has proposed the notion of “epistemic diversity” as a way of understanding these differences. Epistemic diversity, in this context, refers to significant and systematic differences in fundamental concepts, problem formulation, empirical objects, methodological practices, and modes of judgments deployed across the disciplinary spectrum. Methodological factors (including environmental control, statistical inference, precision of research aims, and interpretative flexibility) interact with a host of technical, social, and cultural factors that need to be taken into account when describing conditions for reproducibility across research contexts. A crucial factor for respecting epistemic diversity when mapping these factors is to actively identify those contexts, such as kinds of qualitative research, where reproducibility is less useful (or even problematic) as a general epistemic aim [6]. At the same time, better understanding how concerns and aims are interpreted among other stakeholders (e.g., funders, institutions, and publishers) is also necessary for joint action.

## 3. Systematize evidence for informed policy across contexts

Much is already known about the causes of poor levels of reproducibility. Key issues usually include a lack of preregistration and reporting transparency, a lack of training and Open Science infrastructure, and questionable research practices that are incentivized by publication biases and assessment frameworks that privilege flashy, positive findings [7]. Yet, as is the case with epistemic diversity, neither these issues nor their solutions will play out equally across different disciplines, research cultures, regions, and stakeholders. More experimentation across, between, and even within these contexts should be encouraged to generate comparative findings that can inform interventions. This will enable cross-pollination of successful interventions and deeper understanding of potential gains and savings from increased reproducibility across the research enterprise.

## 4. Work together to boost capacity at all levels

Action is not only needed from a range of stakeholders across diverse epistemic, geographic, and stakeholder contexts; it must also happen at different levels of intervention, as conceptualized by Brian Nosek [8]. We need new tools (infrastructures and services) to enable practices, better interfaces that are intuitive, communities to make them the norm, revised incentives to reward them, and policies to encourage or enforce them as necessary. Great work is already underway across these dimensions, yet more could be done to link and broaden initiatives. How can we better link the rapidly growing web of Reproducibility Networks (peer-led national consortia) to publisher and funder groups, or to large-scale infrastructure efforts such as the European Open Science Cloud? Better coordination of these efforts should be a priority.

## 5. Emphasize inclusion to minimize unintended consequences and maximize equitable transition

Not all impacts will be positive, and trade-offs and unintended consequences are to be expected. My most recent work within the project ON-MERRIT has been concerned with the ways our most well-intentioned efforts to reform research can have negative consequences, especially for the equity of the scientific system [9]. Special attention should be paid not only to the ways that variance in epistemic diversity alters what is desirable in terms of reproducibility, but also to the levels of advancement in dealing with these issues across these contexts. As we rush to reform, we must ensure that policies reflect this diversity, and harness openness of infrastructures, tools, services, and training to move as a global community. The ON-MERRIT final recommendations may help in this regard [10].

## The way ahead

Improving reproducibility requires combined efforts. For my part, I am thrilled to say that I will spend the next few years putting these ideas into action as Project Coordinator and Principal Investigator of TIER2, a new EC-funded project to improve reproducibility across the diverse contexts described here. We will use cocreative methods to work with researchers in social, life, and computer sciences, as well as research funders and publishers, to systematically investigate reproducibility across epistemically diverse contexts, producing and testing new tools, networking initiatives, engaging communities, implementing interventions and policies to increase reuse and overall quality of research results.

On behalf of our consortium, I am excited to invite the broader research community (particularly researchers in our target domains of social science, life science, and computer science, as well as funders and publishers) to work with us to cocreate a reproducibility reformation.
